# Evolution of the Insulin Gene: Changes in Gene Number, Sequence, and Processing

**DOI:** 10.3389/fendo.2021.649255

**Published:** 2021-04-02

**Authors:** David M. Irwin

**Affiliations:** ^1^ Department of Laboratory Medicine and Pathobiology, University of Toronto, Toronto, ON, Canada; ^2^ Banting and Best Diabetes Centre, University of Toronto, Toronto, ON, Canada

**Keywords:** insulin, gene duplication, evolution, adaptive evolution, vertebrates, proteolytic processing, virus

## Abstract

Insulin has not only made major contributions to the field of clinical medicine but has also played central roles in the advancement of fundamental molecular biology, including evolution. Insulin is essential for the health of vertebrate species, yet its function has been modified in species-specific manners. With the advent of genome sequencing, large numbers of insulin coding sequences have been identified in genomes of diverse vertebrates and have revealed unexpected changes in the numbers of genes within genomes and in their sequence that likely impact biological function. The presence of multiple insulin genes within a genome potentially allows specialization of an insulin gene. Discovery of changes in proteolytic processing suggests that the typical two-chain hormone structure is not necessary for all of inulin’s biological activities.

## Introduction

Insulin is well-characterized as a key regulator of blood glucose levels in vertebrates (1). Insulin related peptides have also been identified in a number of other metazoan species and have been shown to contribute to various aspects of physiology in these species (2–4). The discovery of insulin 100 years ago, led to a revolution in clinical medicine, as it allowed an effective treatment for diabetes (5). Since its discovery, the treatment of diabetes using insulin, derivatives of insulin, and other peptides has and continues to evolve (6). There still is no cure. In addition to its critical role in the history of clinical medicine, insulin has played key roles in the development of many revolutionary technologies that are now commonplace in molecular biology, including protein sequencing (7) and the deduction of the three-dimensional structures of proteins (8). A key discovery made with insulin, but with important implications for many other bioactive peptides, is the role of proteolytic processing in regulating its biological action (9, 10). Since the sequencing of human insulin more than 60 years ago (7), a large number of insulin protein sequences have been determined due to its importance in medicine, as well as its small size and relative ease at isolation (11, 12). Over the past 20 years, as we entered the genomic era, an increasing number of insulin sequences have been predicted from the complete genome sequences of organisms. Genomic sequences have led to improvements of our understanding of not only human genetics and disease (13, 14), but also nearly all other areas of biology (15). The new insulin sequences identified from genome sequences have revealed an increased diversity in the number of insulin genes within species and has revealed that changes in the proteolysis processing of the proinsulin precursor likely contributes to the diversity of the biological actions of insulin.

## Superfamily of Insulin-Like Genes

**Figure 1:**
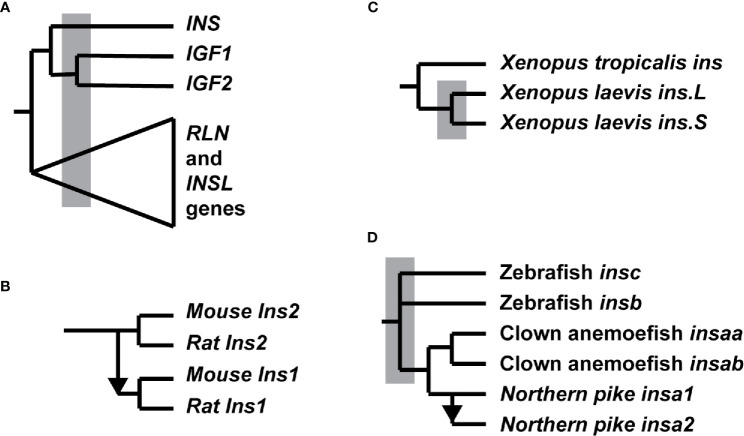
Duplication of insulin-like genes in vertebrates. **(A)** Phylogeny of the insulin supergene family members inferred from their sequences and their genomic locations (18–21). The divergence of the multiple human relaxin (*RLN1*, *RLN2*, and *RLN3*) and insulin-like peptide (*INSL3*, *INSL4*, *INSL5*, and *INSL6*) are indicated by the triangle. Grey box indicates the two genome duplications (2R) that occurred near the origin of vertebrates. **(B)** Origin of the duplicated rodent insulin genes. The *Ins1* gene originated by a retroposition event, shown by the arrow, in the common ancestor of the mouse (*Mus musculus*) and rat (*Rattus norvegicus*), while *Ins2* is located at the locus-of origin (22–26). **(C)** Duplication of the insulin gene in the frog *Xenopus laevis* (27). A pair of insulin genes, *ins.L* and *ins.S*, are found in the *Xenopus laevis* genome due to a genome duplication that has occurred since its divergence from the diploid frog *Xenopus tropicalis*. The grey box indicates the genome duplication. **(D)** Summary of the duplications of insulin genes in bony fish (28). A triplication of the insulin gene, yielding *insa*, *insb*, and *insc* genes, occurred in an early ancestor of teleost fish (the order of the duplications events yielding the *insa*, *insb*, and *insc* is unresolved). These duplications occurred at about the same time as the fish-specific genome duplication (the genome duplication is shown as a grey box) – it is unclear whether if any of the insulin gene duplications were due to the genome duplication. A latter duplication of the *insa* gene in an ancestor of a subset of teleost fish resulted in an *insaa* and an *insab* gene in many fish (e.g., clownfish; *Amphiprion ocellaris*). Insulin genes within some fish genomes also originated *via* retroposition, as indicated by the arrow, with the Northern pike (*Esox lucius*) *insa2* gene being an example.

While insulin was first identified in mammals, it soon became evident that peptides with sequences similar to insulin can be found in diverse multicellular animals, including many non-vertebrate species such as insects and worms (2–4). In many of these species, the insulin-like peptides were found through directed efforts to identify peptides with similarity to insulin, but increasingly, they are now being reported from searches of genome sequences. Multiple insulin-like genes have been characterized in the genomes of many non-vertebrate species that are due to lineage-specific gene duplication events (3, 4). A parallel set of duplications of insulin-like genes has also occurred within vertebrates. In addition to the insulin gene (*INS*), nine other genes encoding peptides with similarity to insulin both in their primary sequences and secondary structures have been identified in the human genome, including 2 insulin-like growth factors (*IGF1* and *IGF2*), 4 insulin-like factors (*INSL3*, *INSL4*, *INSL5*, and *INSL6*), and three relaxins (*RLN1*, *RLN2*, and *RLN3*) (16, 17). While the relationships among these genes cannot be fully resolved by phylogenetic analysis, due to their short protein lengths, information derived from their locations within the genome has helped to largely resolve the order and timing of the gene duplication events that generated this gene family (18–21). As summarized in **Figure 1A**, these studies suggest that the initial gene duplication event that originated this gene family separated an ancestor for the insulin and insulin-like growth factor (*IGF1* and *IGF2*) genes from an ancestor of the insulin-like (*INSL*) and relaxin (*RXN*) genes. This duplication was then followed by a duplication that separated the insulin gene from an ancestor of the insulin-like growth factor (*IGF1* and *IGF2*) genes. Both of these gene duplication events occurred before the two genome duplications that are associated with the origin of vertebrates (18).

The human insulin gene (*INS*) is a small gene of 1,425 base pairs located on chromosome 11 and is composed of 3 exons separated by two introns (29). The first exon of this gene is composed entirely of 5’ untranslated sequence, with all of the coding region, which encodes the 110 amino acid long proinsulin precursor protein, distributed across exons 2 and 3 (29). The N-terminal portion of the proinsulin precursor protein sequence is a signal peptide that allows secretion from pancreatic beta cells and is removed by signal peptidase to yield proinsulin (10, 30). Further processing by prohormone convertase enzymes releases the internal C-peptide to produce the two-chain insulin hormone, composed of A- and B-chains linked by disulphide bridges (10, 30). The proteolytic processing of proinsulin to yield a two-chain insulin hormone contrasts with that of the insulin-like growth factors, where both are single chain peptides that retain the C-peptide-like sequences (31, 32). Insulin genes from diverse species representing multiple classes of vertebrates, including fish, frogs, and birds, have been characterized that share with the human gene a similar three exon (two coding) gene structure, and encode homologous protein sequences (30, 33). In most vertebrates only a single insulin gene has been found, however, multiple copies of this gene, including some with differing gene structures, have been found in some species.

## Duplicated Insulin Genes

Rats (*Rattus norvegicus*) and mice (*Mus musculus*) were the first vertebrates found to each have two insulin proteins, which were subsequently found to be encoded by a pair of genes (22). While the insulin 2 genes (*Ins2*) have a gene structure similar to that of the human gene (three exons and two introns), the insulin 1 gene (*Ins1*) was found to be composed of only 2 exons, with all of the coding sequence contained in the second exon (23). These two genes have a relatively recent origin (see **Figure 1B**), in the common ancestor of mice and rats (22–26). Further study of these genes indicated that the *Ins1* gene originated from an aberrant mRNA transcript that was initiated about 500 bases upstream of the normal mRNA start site and partially processed to remove only intron 2 sequences before being reverse transcribed and inserted into the genome (23, 24, 26). Despite the differences in the structures of these two genes, both are equally expressed (22, 24). Since the mouse and rat *Ins1* genes only have ~500 bases of 5’ flanking sequence homologous to the *Ins* genes of other mammals, this indicates that only a limited amount of sequence is needed for efficient beta-cell-specific expression of the insulin gene. But why do mice and rats have two insulin genes that have identical expression patterns, while other species can survive with a single gene? This might suggest that the two genes differ in function, perhaps specializing function at different sites or times, thus generating a need to retain both genes. While some evidence for differences in the selective constraints acting on the two insulin genes has been detected (25), no evidence for different functions have been found, thus a convincing explanation has not been reached. Intriguingly, duplicated insulin genes that originated by retroposition, like the rodent *Ins1* gene, have also been described in several species of bony fish through three independent origins (28). While most of the retroposed insulin-like sequences in fish appeared to be pseudogenes, the gene sequence of a potentially retroprocessed insulin gene in the Northern pike (*Esox lucius*) has an intact coding sequence (**Figure 1C**) that is potentially functional (28). Further study is needed to determine whether this retroposed Northern pike insulin gene is expressed and if its encoded protein has a physiological function. These observations also indicate that the vertebrate insulin gene is expressed in the germ cells of a number of species, thus allowing it to be retroprocessed and integrated into the genome allowing to be passed on to the next generation, raising questions about the possible function of insulin in germ line cells.

Duplicated copies of the insulin gene have also been found in several other vertebrate species (**Figures 1C, D**), where these genes retain the three exon and two intron gene structure and potentially have large amounts of flanking sequences that would allow their continued expression. The frog *Xenopus laevis*, which experienced a recent genome duplication (**Figure 1D**), was the first published example (34). Unlike the duplicated rodent insulin genes, the two *Xenopus laevis* insulin genes were found to display differing developmental gene expression patterns that might suggest diverging functions (34), and a reason why both are retained, however they also had overlapping gene expression in the adult pancreas (35). A genome duplication, the fish-specific whole genome duplication (3R), was experienced by an ancestor of teleost fish (36), thus the discovery of a second insulin gene in several fish genome sequences (37) was not a surprise. Characterization of the two zebrafish (*Danio rerio*) insulin genes (*insa* and *insb*) provided evidence that the two genes had distinct expression patterns and potentially differing functions (38), supporting a hypothesis that fish genes have diverged in function, potentially subfunctionalizing so that each now is responsible for a subset of the ancestral functions of insulin. More recently, a third insulin gene (*insc*) has been found in some, but not all, fish (**Figure 1C**), with all three of these genes originating in an early teleost, thus it is unclear which, if any, originated through the fish-specific whole genome duplication (28). The role of this third insulin gene in fish physiology is unknown. Does it also possess a subset of ancestral insulin functions, or has it gained new function? In addition to these three types of insulin genes found in fish, additional lineage-specific duplications of insulin genes were found, including some species [e.g., carp (*Cyprinus carpio*)] that are associated with additional genome duplications on these lineages (28). In addition to these lineage-specific duplications due to genome duplications, a duplication of the *insa* insulin gene, resulting in the *insaa* and *insab* genes, occurred early in the diversification of teleost fish (**Figure 1C**) yielding a large number of species with these gene duplicate. Intriguingly, most of the proteins encoded by the *insab* genes have amino acid substitutions that are predicted to impair proteolytically processing that generates the typical two-chain insulin hormone (28). While these sequences retain signal peptides, which would allow secretion, and cysteine residues that allow disulphide bridge formation, this raises the possibility that they yield an unprocessed inulin-like protein that retains an insulin-like protein structure and has an unknown function (28).

## Evolution of Insulin Sequences

In addition to changes in the numbers and structure of insulin genes, sequences of insulin genes have also changed. Typically, genes evolve at a near steady rate, but occasionally they display episodes of more rapid change, which are hypothesized to signal a change in gene function. Studies of mammalian insulins have provided support for this hypothesis. Insulin sequences from the guinea pig (*Cavia porcellus*) and relatives (rodents of the suborder Hystricomorpha) are well known for having insulin sequences with highly divergent sequences (39, 40). The biological activities of these insulins also differ, acting more as a growth factor than as a metabolic hormone (41, 42). These changes, in sequence and function, have been accompanied with an acceleration of the rate of evolution of the insulin protein sequence in the guinea pig and relatives (40, 43, 44). Similar, but less dramatic, episodes of accelerated evolution of insulin sequences have been observed in some species of New World monkeys (44, 45), species that have insulin hormones with lower potency (46).

## Changes in Proteolytic Processing

**Figure 2:**
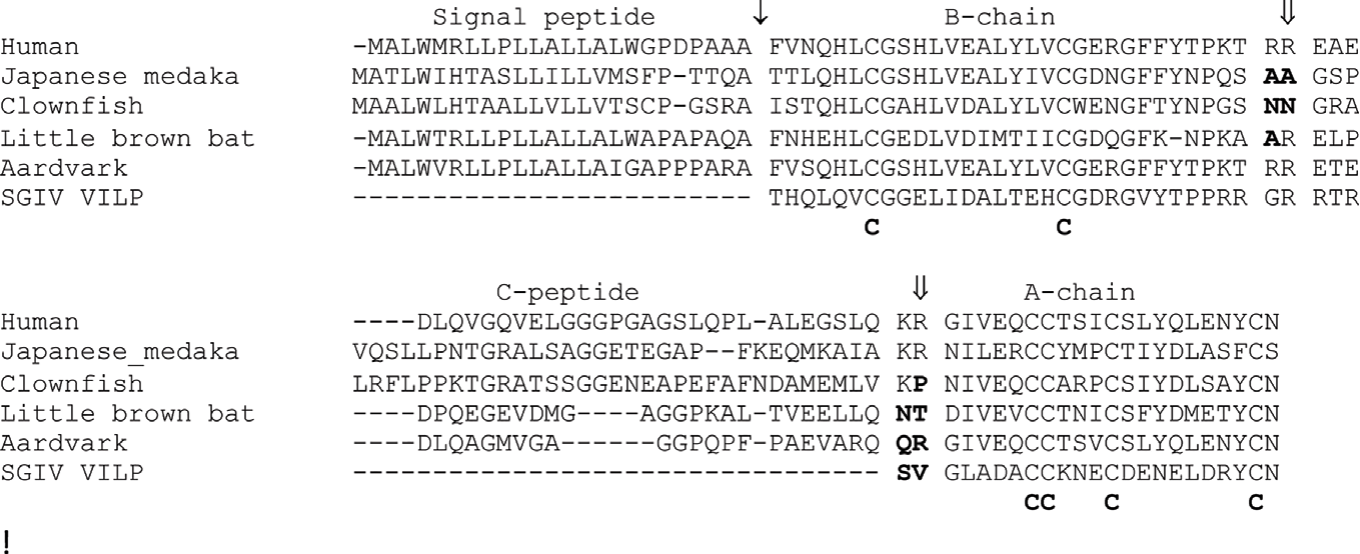
Changes in in the processing of proinsulin-like sequences found in vertebrates. An alignment of the human (*Homo sapiens*) proinsulin protein sequence with selected examples showing potentially altered proteolytic processing. The examples include the insulin proteins encoded by the *insab* genes from two fish (28) [Japanese medaka (*Oryzias latipes*) and Clownfish (*Amphiprion ocellaris*)], two mammals (44) [little brown bat (*Myotis lucifugus*) and aardvark (*Orycteropus afer*)], and the Singapore grouper iridovirus viral insulin-like peptide (SGIV-VILP) (51). SGIV-VILP would be produced by vertebrate cells infected by the Singapore grouper iridovirus. Protein sequences are shown in single letter code, with ↓ indicating the signal peptidase cleavage site and ⇓ the indicating the prohormone convertase processing sites for the human insulin sequence. Domains of the human proinsulin sequence are shown above the alignment. Amino acid replacements in the sequences, relative to the human sequence, which are predicted to impair proteolytic processing are shown in bold. Conserved cysteine residues involved in disulphide bridging are shown below the alignment.

Recent surveys of fish and mammalian insulin coding sequences have identified several species that have accumulated increased amounts of sequence change (28, 44). However, in contrast to the sequences from the rodent suborder of Hystricomorpha and New World monkeys, the striking changes in these sequences were at sites involved in proteolytic processing. Insulin is composed of two peptide chains linked by disulphide bonds, with both peptide chains generated from a single precursor protein (9, 10). Studies on insulin emphasized the importance of proteolytic processing in the generation of bioactive peptides (2, 9, 10, 47). The insulin precursor undergoes two types of proteolytic processing to generate a functional hormone: 1) removal of its signal peptide, which is necessary for secretion, and 2) removal of the C-peptide to generate the two-chain molecule linked by disulphide bonds (10, 48). Evolutionary changes have occurred at all of these proteolytic sites. Changes in the sites of proteolytic processing of the insulin precursor had previously been observed, especially at the signal peptidase cleavage site and at the B-chain/C-peptide processing site (12). While some earlier studies have suggested altered proteolytic processing of proinsulin in some species of the most divergent classes of vertebrates, jawless and cartilaginous fish (49, 50), recent studies of insulin gene sequences obtained from the genomic sequences of some species of bony fish and mammals predict amino acid replacements that abolish proteolytic processing at both the B-chain/C-peptide and the C-peptide/A-chain processing sites of insulin (28, 44). Almost all of the proinsulin sequences predicted by teleost fish *ins* genes encode sequences that can be processed into two-chain insulin hormones, except for those encoded by *insab* genes (28). Most proinsulin sequences encoded by the *insab* genes contain amino acid substitutions at their B-chain/C-peptide processing site that likely prevent proteolytic cleavage, with many of them also having substitutions that should impair processing at the C-peptide/A-chain site (**Figure 2**), thus leading to improperly processed insulin molecules (28). Similarly, insulin gene sequences from two bats (*Myotis brandtii* and *M. lucifugus*) predict substitutions at both the A-chain/C-peptide and C-peptide/B-chain processing sites that should prevent processing (**Figure 2**), while the insulin sequences from several species of Afrotheria [e.g., aardvark (*Orycteropus afer*)] likely have altered C-peptide/A-chain processing (**Figure 2**) (44). Thus, it is likely that the insulin genes of many vertebrate species generate a final protein product that is composed of either a single protein chain or are two-chain protein molecules that have an A- or a B-chains that is extended to include the complete C-peptide sequence (28, 44). Intriguingly, all of these insulin sequences with altered protein processing retain conserved cysteine residues that are important for disulphide bridging, thus, these proteins potentially retain three-dimensional structures that are similar to insulin and are also biologically functional.

## Viral Insulin-Like Peptides

Most studies on the function of insulin assume that this peptide is of endogenous origin, or from relatively closely related species. Indeed, humans have been treated with insulin from several other mammalian sources (5). Recent studies analyzing the sequences of viral genomes have revealed that some viruses that infect vertebrates could be another source of insulin-like peptides, with these peptides having the potential to affect physiology and pathophysiology (51). Altindis et al. (51) identified four viruses, which infect fish, whose genomes predict peptides with similarity with insulin, which they called viral insulin-like peptides (VILPs). While these new VILPs sequences share similarity with insulin, differences exist at the regions corresponding to the B-chain/C-peptide and C-peptide/A-chain processing sites (**Figure 2**), thus, and might not generate two-chain molecules (51). However, like the incompletely processed vertebrate insulin sequences described above, the VILPs share the conserved cysteine residues involved in disulphide bridging and are requisite to the maintain the 3D structure (51). Some VILPs have been shown to bind to insulin receptors and regulate glucose metabolism in mice (51, 52), indicating that it may function in its proinsulin form, and that they might have a pathophysiological role beneficial to the viruses. Insulin-like molecules are used as toxins by cone snails (53), thus the use of an insulin-like peptide by a virus in pathophysiology should not be a surprise. It has long been known that full length proinsulin can bind and activate the insulin receptor (54), as do the single-chain IGF1 and IGF2 hormones with their specific receptors, thus, the incompletely processed insulin molecules encoded by genes in fish, mammals, or viruses could impact physiology or pathophysiology.

## Perspectives and Future Directions

The biology of insulin as well as the evolution of insulin have been studied for many years (2, 9, 12) yet new discoveries and insights have been gained from the analysis of the rapidly increasing amount of genomic data. Studies into the evolution of the genes for the insulin receptor, enzymes involved in producing the mature hormone and downstream signaling partners should also improve our understanding of the biology of insulin. Genomic sequences, together with improved bioinformatic search algorithms, allow unbiased searches for sequences with similarity to insulin (or your favorite protein) in genomes, revealing sequences that might not have been found in more directed searches for bioactive peptides. As we complete more genomes and microbiomes, it is certain that we will discover more insulin-like sequences with novel aspects to their sequences, structures, and functions. However, sequence will not tell us function. Experimental work is still needed to identify the functions of these novel insulin-like sequences, which may uncover new roles for insulin in biology.

## Author Contributions

The author confirms being the sole contributor of this work and has approved it for publication.

## Conflict of Interest

The author declares that the research was conducted in the absence of any commercial or financial relationships that could be construed as a potential conflict of interest.
